# Spatial Factors Outweigh Environmental Variables in Explaining Fish Beta Diversity Within A Massive Anthropogenically Homogenized Water‐Transfer Canal

**DOI:** 10.1002/ece3.74077

**Published:** 2026-07-29

**Authors:** Zhibin Song, Jia Li, Min Zhang, Zhihai Li, Zihua Qu, Haohan Yuan, Xiaodong Qu, Baohang Zhang, Chengwei Chen, Xiaobo Liu

**Affiliations:** ^1^ State Key Laboratory of Water Cycle and Water Security China Institute of Water Resources and Hydropower Research Beijing China; ^2^ College of Hydrology and Water Resources Hohai University Nanjing China; ^3^ Bureau of South to North Water Transfer of Planning, Designing and Management, Ministry of Water Resources Beijing China; ^4^ Henan Branch China South‐to‐North Water Diversion Corporation Limited Zhengzhou China

**Keywords:** beta diversity, dispersal limitation, food resources, habitat homogenization, inter‐basin water transfer project

## Abstract

Inter‐basin water transfer projects serve as critical artificial corridors that optimize the allocation of water resources. Understanding their influence on aquatic community assembly is a frontier issue. This study focused on the Middle Route of the South‐to‐North Water Diversion Project, the world's largest water transfer project. By integrating eDNA technology with functional trait analysis, we systematically investigated the spatial patterns of fish taxonomic and functional beta diversity in this highly homogenized artificial habitat. In the present study, 39 fish taxa were identified, with significant spatial variations in community structure. A distinct functional trait succession gradient emerged with increasing latitude, characterized by a trend towards smaller‐bodied communities and feeding shifts towards piscivores and invertivores. Both taxonomic and functional beta diversity were primarily driven by turnover components and exhibited significant positive spatial correlations. However, functional beta diversity lagged significantly behind taxonomic beta diversity numerically. This synchronous but non‐isometric pattern indicates functional convergence induced by the artificial habitat, suggesting potential risks of functional redundancy loss under intense human intervention. Variance partitioning showed spatial factors outweighed environmental factors in shaping beta diversity. This suggests that in physically unobstructed and homogenized artificial ecosystems, dispersal limitation driven by geographic distance and unique hydraulic conditions outweighs environmental filtering as the primary deterministic process shaping diversity patterns. Furthermore, the inclusion of food resource variables increased the explanatory power of the model, suggesting that spatial variation in resource availability may contribute to fish community structure. The findings indicate that management of artificial water diversion systems should transcend conventional water quality monitoring. Strengthening biosecurity surveillance and implementing functional compensatory measures at critical habitat nodes will be essential to offset the “ecological distance” imposed by dispersal limitation. This approach ensures the long‐term functional stability of ecosystems and safeguards water supply security within massive anthropogenically homogenized water diversion projects.

## Graphical abstract

In physically unobstructed and homogenized artificial ecosystems, dispersal limitation driven by geographic distance and unique hydraulic conditions replace environmental filtering as the primary deterministic process shaping diversity patterns. Furthermore, significant regulatory effects of food resources through trophic cascades reinforce this spatial community assembly based on resource accessibility.

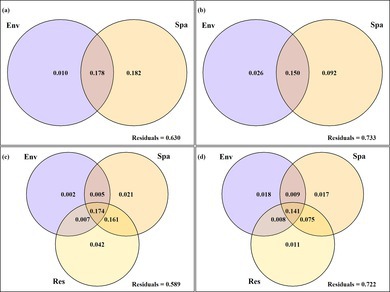

## 1 Introduction

Inter‐basin water transfer projects (IBWTs) serve as critical infrastructure for addressing the spatial imbalance of global water resources. However, as large‐scale artificial corridors, their reshaping effects on aquatic biodiversity and ecosystem functions have drawn widespread attention from the international ecological community. By physically connecting watersheds, these projects break geographical isolation, inducing directed dispersal and community reassembly (Hu et al. 2024; Zhang, Bi, et al. 2023). However, compared to natural river ecosystems, the main canals of IBWTs typically exhibit highly homogenized habitat characteristics and controlled hydraulic conditions. Significant theoretical debate persists regarding how such intense human intervention alters the mechanisms of fish community assembly.

As consumers occupying a variety of high‐trophic positions in freshwater ecosystems, fish play a critical role in maintaining the structural integrity and functional stability of aquatic food webs (Xie et al. 2025; Zhao et al. 2025). Through top‐down effects, fish significantly influence the population dynamics and spatial distribution of plankton, macroinvertebrates, and benthic algae (Benkendorf and Whiteman 2021; Zhang, Shen, et al. 2023). Consequently, the composition and functional traits of fish communities not only integrate long‐term shifts in the physicochemical environment, but also serve as comprehensive biological indicators of energy flow and material cycling within river basins. In highly simplified artificial ecosystems like the Middle Route of the South‐to‐North Water Diversion Project, fish as apex predators often exhibit heightened sensitivity to fluctuations in bottom‐dwelling resources due to highly low habitat heterogeneity and the absence of complex intermediate trophic level buffers. By examining fish diversity patterns, we can capture the coupling relationships among trophic levels within artificial canal food webs at the macro‐scale, providing crucial evidence for assessing the ecological health of the water diversion project ecosystem.

Given the vital role of fish within food webs, unraveling the community assembly mechanisms is central to studying the ecological effects of water diversion projects. Community assembly theory posits that the spatial patterns of biodiversity result from the combined effects of environmental filtering, dispersal limitation, and random processes (Astorga et al. 2014; Chust et al. 2013; Heino et al. 2015). In natural river ecosystems, environmental filtering is typically regarded as the dominant force shaping fish beta diversity (Heino et al. 2017; Lopez‐Delgado et al. 2020). However, within the highly homogenized habitat of artificial canals, the attenuation of environmental gradients may weaken the strength of environmental filtering, potentially allowing spatial processes—often interpreted as a proxy for dispersal limitation—to play a more prominent role. Moreover, conventional analyses focused solely on taxonomic diversity often fail to capture the continuity of ecosystem function. Incorporating functional traits into beta diversity research offers a more incisive lens through which to assess how communities respond to environmental change. As Villeger et al. (2013) pointed out, the decoupling or synchronization of taxonomic and functional beta diversity directly serves as a powerful indicator of functional redundancy and stability of ecosystems.

The Middle Route of the South‐to‐North Water Diversion Project, the largest inter‐basin water transfer project in the world, spans the Yangtze River, Huaihe River, Yellow River, and Haihe River basins with its main canal. Stretching over 1000 km, this fully lined artificial watercourse constitutes a unique “macro‐scale experimental arena,” characterized by high hydrological connectivity but extreme habitat homogenization. Previous research has predominantly focused on water quality dynamics (Nong et al. 2020; Tang et al. 2022; Wang et al. 2023) or shifts in taxonomic composition (Wang et al. 2025; Zhang, Bi, et al. 2023). Surprisingly little attention, however, has been devoted to understanding the spatial coupling between taxonomic and functional diversity or to the multidimensional drivers. In particular, quantitative evidence is lacking on how trophic mediators such as macroinvertebrates, plankton, and benthic algae synergize with spatial and environmental factors to reshape the distribution patterns of high‐trophic‐level fish species.

This study employs environmental DNA (eDNA) metabarcoding to monitor fish diversity along the Middle Route's main canal, aiming to address the following scientific questions: (1) To analyze the spatial patterns of fish taxonomic diversity and functional beta diversity within the artificial canal, and to determine whether functional convergence or functional lag occurs; (2) Quantitatively assess the relative contributions of spatial factors, environmental factors, and resource availability to community assembly through variance partitioning analysis. We hypothesize that due to the highly homogenized habitat of the canal, spatial factors—used here as a proxy for dispersal limitation—will explain a larger proportion of variation in fish community composition than the measured environmental variables. Furthermore, the distribution of resources will significantly enhance the model's explanatory power for spatial community reassembly through resource‐associated effects. Our findings will provide new insights into the assembly patterns of aquatic communities under intense human intervention and offer theoretical foundations for the ecological safety management of IBWTs.

## 2 Materials and Methods

### 2.1 Study Area

The Middle Route of the South‐to‐North Water Diversion Project originates at the Danjiangkou Reservoir, traversing Henan and Hebei provinces before terminating in the municipalities of Beijing and Tianjin. Its geographical extent spans latitudes 32.68° N to 39.99° N and longitudes 111.72° E to 116.27° E. Stretching over 1000 km, it traverses three monsoon climate zones (subtropical, warm‐temperate, and temperate), and is one of the largest inter‐basin water transfer projects globally (Wang et al. 2023; Zhang, Bi, et al. 2023). The main canal is entirely lined with concrete and operates as a closed system, incorporating various hydraulic structures such as open canals, aqueducts, inverted siphons, culverts, and tunnels. Consequently, the habitat exhibits high homogenization and remains largely hydrologically isolated from adjacent natural water bodies, forming an independent artificial water conveyance ecosystem (Du et al. 2021; Ma et al. 2025). This project significantly alleviates water scarcity in North China, supplying domestic, industrial, and agricultural water to over 20 large and medium‐sized cities along its route, benefiting more than 100 million people (Nong et al. 2023; Wang et al. 2023).

**FIGURE 1:**
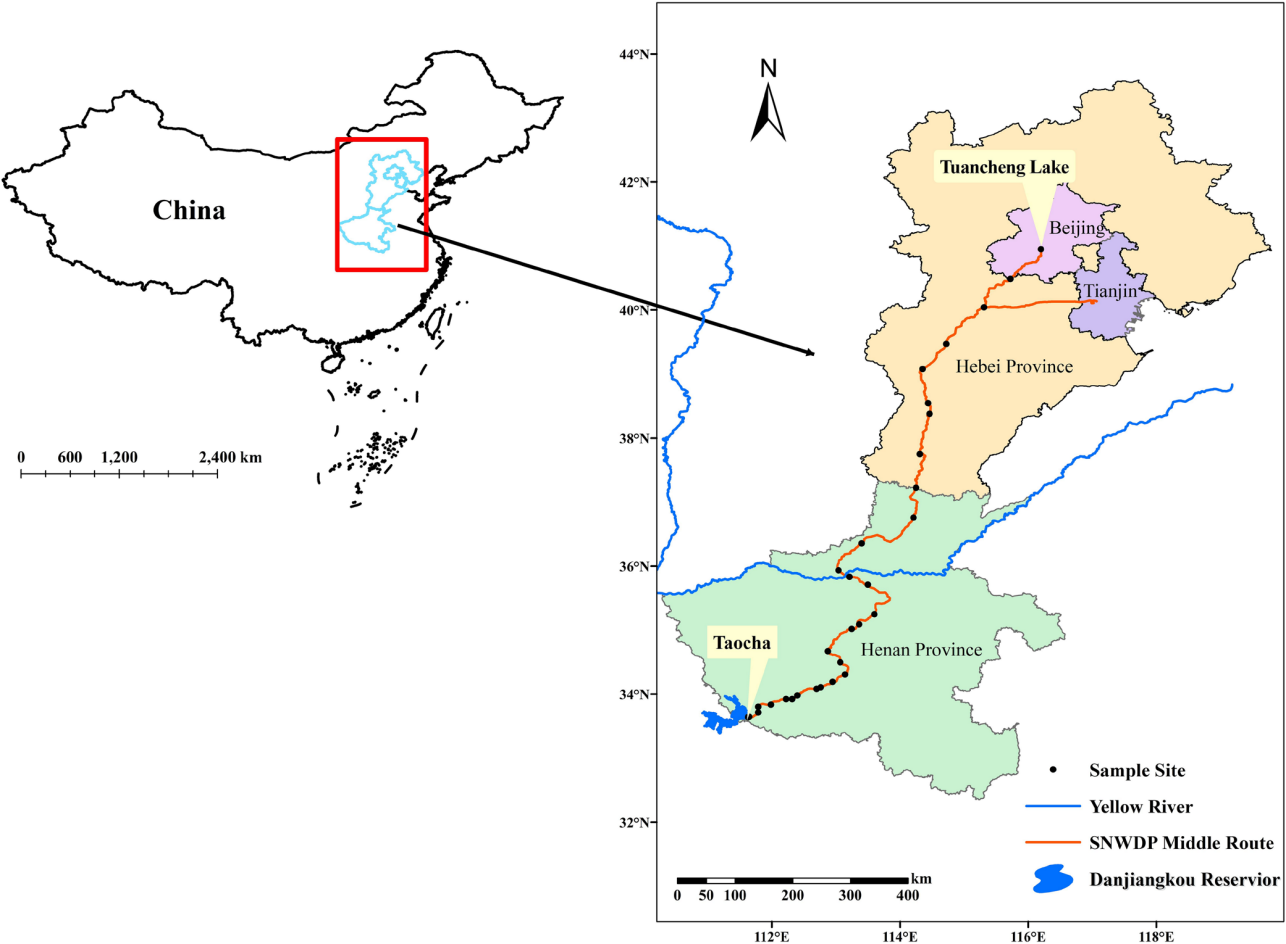
Sampling sites along the Middle Route of the South‐to‐North Water Diversion Project.

Field sampling for this study was conducted between April and June 2025, targeting key biotic compartments: fish, macroinvertebrates, plankton, and benthic algae. A total of 31 sampling sites were established from the Taocha Headwork Management Office to the Tuancheng Lake in Beijing. Among these, 22 sites were located in Henan Province, seven in Hebei Province, one in Beijing, and one in Tianjin. Because Beijing and Tianjin each had only one sampling site, which precluded independent statistical analysis, they were combined with Hebei Province into a single “Hebei (including Beijing and Tianjin)” group for comparative analysis. This grouping is justified by their geographical continuity along the Middle Route of the South‐to‐North Water Diversion Project. The spatial distribution of these sampling locations is illustrated in Figure 1.

### 2.2 Sample Collection and Sequencing

For fish, two 5 L surface water samples (0.3–0.6 m below the water surface) were collected from each sampling site using sterile water bags. Samples were transported to the laboratory under refrigerated conditions and filtered through 0.45 μm mixed‐cellulose ester membranes (Millipore, USA) within 12 h. The membranes were stored at −20°C until DNA extraction.

DNA extraction was performed using a commercial water DNA extraction kit (DNeasy PowerWater Kit, Qiagen, Hilden, Germany) following the manufacturer's protocol, with the following modifications: the filter membrane was cut into small pieces and incubated with 500 μL of lysis buffer at 56°C for 30 min, and DNA was eluted in 100 μL of TE buffer. One extraction blank (sterile ultrapure water processed through the entire extraction procedure) was included per batch to monitor cross‐contamination.

PCR amplification targeted the fish mitochondrial 12S rRNA gene using the universal MiFish‐U/E primer pair: MiFish‐U/E‐F (5′‐GTYGGTAAAWCTCGTGCCAGC‐3′) and MiFish‐U/E‐R (5′‐CATAGTGGGGTATCTAATCCYAGTTTG‐3′). Each 50 μL PCR reaction contained 25 μL of 2× PrimeStar Max DNA Polymerase (Novoprotein, China), 2 μL of each primer (10 μM), approximately 50 ng of template DNA, and nuclease‐free water to 50 μL. The thermal cycling conditions were: initial denaturation at 98°C for 30 s; 38 cycles of 98°C for 10 s, 58°C for 30 s, 72°C for 20 s; and a final extension at 72°C for 5 min. PCR negative controls (ultrapure water instead of DNA template) and field blank (ultrapure water processed through filtration and extraction) were included in each batch. Three technical replicates were performed per sample and pooled prior to library preparation. No positive control or mock community was used (Ardura et al. 2010). Throughout the procedure, masks and gloves were worn, and all experimental equipment was sterilized to prevent exogenous contamination.

Throughout the procedure, masks and gloves were worn, and all experimental equipment was sterilized to prevent exogenous contamination.

For benthic algae, three parallel samples were scraped from a fixed area (0.0625 m^2^) on the canal wall at each sampling site. These were diluted to a fixed volume in 1000 mL sample bottles. Immediately after collection, 60 mL algal suspension was transferred to specimen bottles and preserved with 4% formalin for subsequent taxonomic identification (Hu et al. 2022). Macroinvertebrates were quantitatively sampled using a Surber net (mesh size: 0.42 mm; sampling area: 0.0625 m^2^). At each site, three parallel samples were collected and subsequently pooled into a single composite sample, which was preserved in the field with 95% ethanol. In the laboratory, all samples were sorted, and individuals were identified and counted following standard taxonomic references (He et al. 2020). For phytoplankton, a water sample (1 L) was taken within the upper 0.5 m of the water column and preserved on site by the addition of acidic Lugol's solution. After allowing the sample to settle for 48 h, the supernatant was carefully removed by siphoning, concentrating the sample to approximately 60 mL. This concentrate was then preserved with 4% formalin for later phytoplankton identification (Wang et al. 2025). Zooplankton was collected by filtering 30 L of water through a plankton net (26 μm), and the retained material was preserved with 4% formalin (Li et al. 2022).

Dissolved oxygen (DO), conductivity (Cond), salinity (Sal), water temperature (WT), and pH were measured using a multi‐parameter water quality analyzer (YSI ProPlus) at each sampling site. Additionally, 1000 mL of water sample was acidified with concentrated sulfuric acid to pH < 2, and stored at low temperature for transport to the laboratory. Laboratory analysis determined water quality parameters including total nitrogen (TN), ammonia nitrogen (NH_3_–N), nitrate nitrogen (${\mathrm{NO}}_{3}^{-}$–N), total phosphorus (TP), silicate (${\mathrm{SiO}}_{3}^{2-}$–Si), total organic carbon (TOC), and chemical oxygen demand (COD_Mn_). Flow velocity (V) was measured using a direct‐reading flowmeter (Global Water FP101) at each sampling site, and the average of three replicate measurements was recorded. The measurements were carried out in accordance with the Environmental Quality Standards for Surface Water (China Environmental Science Press 2002a), the Standard Methods for the Examination of Water and Wastewater (China Environmental Science Press 2002b). Refer to Table S2 for the variation of environmental factors along the entire canal.

### 2.3 Bioinformatic Processing

Library preparation was performed using the ALFA‐SEQ DNA Library Prep Kit for MGI (FINDROP, China) following the standard protocol. Sequencing was carried out on an Illumina NovaSeq 6000 platform (Illumina, USA) with a paired‐end 250 bp (PE250) read length.

Raw sequencing data underwent quality control using Fastp software, removing sequences shorter than 100 bp, with N base proportions exceeding 5%, or average quality scores below 20. USEARCH performed paired‐end sequence assembly, while VSEARCH removed primers and discarded sequences with sequencing error rates exceeding 1%, retaining only those within the amplified fragment length range of 170–190 bp. Potentially human‐derived sequences were removed by alignment against human metabarcoding reference sequences. After pooling all sample assemblies, VSEARCH performed deduplication and retained representative sequences with abundance ≥ 4. Chimerism was then eliminated using the uchime3_denovo algorithm in USEARCH. UPARSE performed OTU clustering at 97% similarity. The resulting OTU representative sequences were BLAST‐aligned against reference databases, discarding sequences with alignment lengths < 90% or similarity < 80%. Taxonomic levels were defined at 98%, 95%, 90%, and 85% similarity thresholds for species, genus, family, and order, respectively (Macher et al. 2021). Additionally, historical fish survey data from this region were integrated as supplementary references for taxonomic identification to enhance species recognition reliability. Rarefaction was performed to normalize read counts across samples, and OTUs with a total read count < 10 across all samples were removed to minimize the influence of sequencing errors.

### 2.4 Selection of Functional Traits

Referencing the functional trait framework proposed by Villeger et al. (2017), five functional traits were selected: body size, body shape, feeding, mature size, and spawning type (Table 1). The classification criteria for each trait were as follows: body size was categorized as small (< 30 cm), medium (30–100 cm), or large (≥ 100 cm); body shape (fusiform, compressiform, or anguilliform) was defined based on body depth‐to‐width ratio and ecological habit, primarily following FishBase; feeding functional groups were assigned according to literature consensus from the study area and adjacent waters, cross‐checked with FishBase; for diet‐flexible species, local diet data were prioritized, and FishBase records were used when local data were unavailable; mature body size (maximum body length, cm) was retrieved from FishBase; missing trait values were imputed using a hierarchical strategy (congeneric > confamilial > conorder), with imputation sources noted in the Tables S1–S6. Spawning type was classified following published regional records. Full details and the complete functional trait matrix for all detected taxa are provided in Data [Link], [Link].

**TABLE 1:** Functional trait framework for characterizing the community structure of fish assemblages.

Functional traits	Functional groups
Body size	Small (Maximum body length < 30 cm)
	Medium (30 cm ≤ Maximum body length < 100 cm)
	Large (Maximum body length ≥ 100 cm)
Body shape	Fusiform
	Cylindrical
	Subcylindrical
	Compressiform
	Depressiform_compressiform
	Cylindrical_compressiform
	Eel
Feeding	Omnivorous
	Herbivorous
	Planktivorous
	Invertivorous
	Piscivorous
Spawning type	Adhesive demersal eggs
	Drifting eggs
	Laying eggs in the gill flap of the mussel
Mature size	Continuous variable

### 2.5 Statistical Analyses

First, generalized linear models (GLMs) were employed to analyze the spatial trends of fish functional traits along the canal. For each functional trait, the community weighted mean (CWM) was used as the response variable, and the along‐canal distance (km) was used as the predictor representing the spatial gradient. A Gaussian distribution with an identity link function was used, and the normality and homoscedasticity of residuals were checked. *p*‐values were adjusted for multiple trait models using the Benjamini–Hochberg false discovery rate (FDR) method.

Non‐metric multidimensional scaling (NMDS) combined with permutational multivariate analysis of variance (PERMANOVA) was used to test differences in fish community structure and functional trait composition between Henan and Hebei provinces. PERMANOVA was performed on Bray–Curtis dissimilarity matrices. Before each PERMANOVA, homogeneity of multivariate dispersions was tested using the betadisper function (999 permutations), and no significant differences were found (*p* > 0.05).

Total beta diversity of fish communities was decomposed into turnover and nestedness components using the framework of Baselga (2010). Both taxonomic beta diversity and functional beta diversity were calculated using the Jaccard index. The decomposition for taxonomic data was performed using the beta.pair and beta.multi functions in the betapart package, and for functional data using the mFD package.

A paired *t*‐test was used to compare the difference between taxonomic and functional beta diversity. The test was performed on the pairwise dissimilarity values to assess whether the two types of beta diversity differed significantly.

Mantel tests were used to analyze correlations between beta diversity and both geographical and environmental distances. Geographical distance was calculated as Euclidean distance (km) from site coordinates (latitude and longitude). Environmental distance was calculated as Euclidean distance from standardized environmental variables (water temperature, pH, nutrients, etc.). All Mantel tests were performed with 9999 permutations to obtain robust *p*‐values.

Variation partitioning was performed within the Multiple Regression on distance Matrices (MRM) framework to quantify the independent and combined explanatory effects of spatial factors, environmental factors, and food resource factors on taxonomic and functional beta diversity. Spatial factors were represented by a Euclidean distance matrix calculated from site coordinates (latitude and longitude). Environmental factors were represented by a Euclidean distance matrix derived from standardized environmental variables (e.g., water temperature, pH, nutrients). For food resource factors, we first calculated separate Bray–Curtis dissimilarity matrices based on relative abundances for each of the four biological groups: macroinvertebrates, phytoplankton, zooplankton, and benthic algae. Then, in the variation partitioning, all four distance matrices were entered together as a single set of predictors (the food resource complex) into the MRM model to parse the unique and shared explanatory effects of each biological group on fish beta diversity. Taxonomic beta diversity was based on the Bray–Curtis dissimilarity matrix of species relative abundances, while functional beta diversity was based on the trait‐weighted Bray–Curtis dissimilarity matrix.

All statistical analyses were performed in R (version 4.5.1). The “vegan” package was used for NMDS, PERMANOVA, and variation partitioning. Taxonomic beta diversity was calculated and partitioned using the “betapart” package, while functional beta diversity was computed and decomposed with the “mFD” package. All figures were generated using “ggplot2.”

## 3 Results

### 3.1 Taxonomic Composition and Spatial Patterns of Fish Assemblages

**FIGURE 2:**
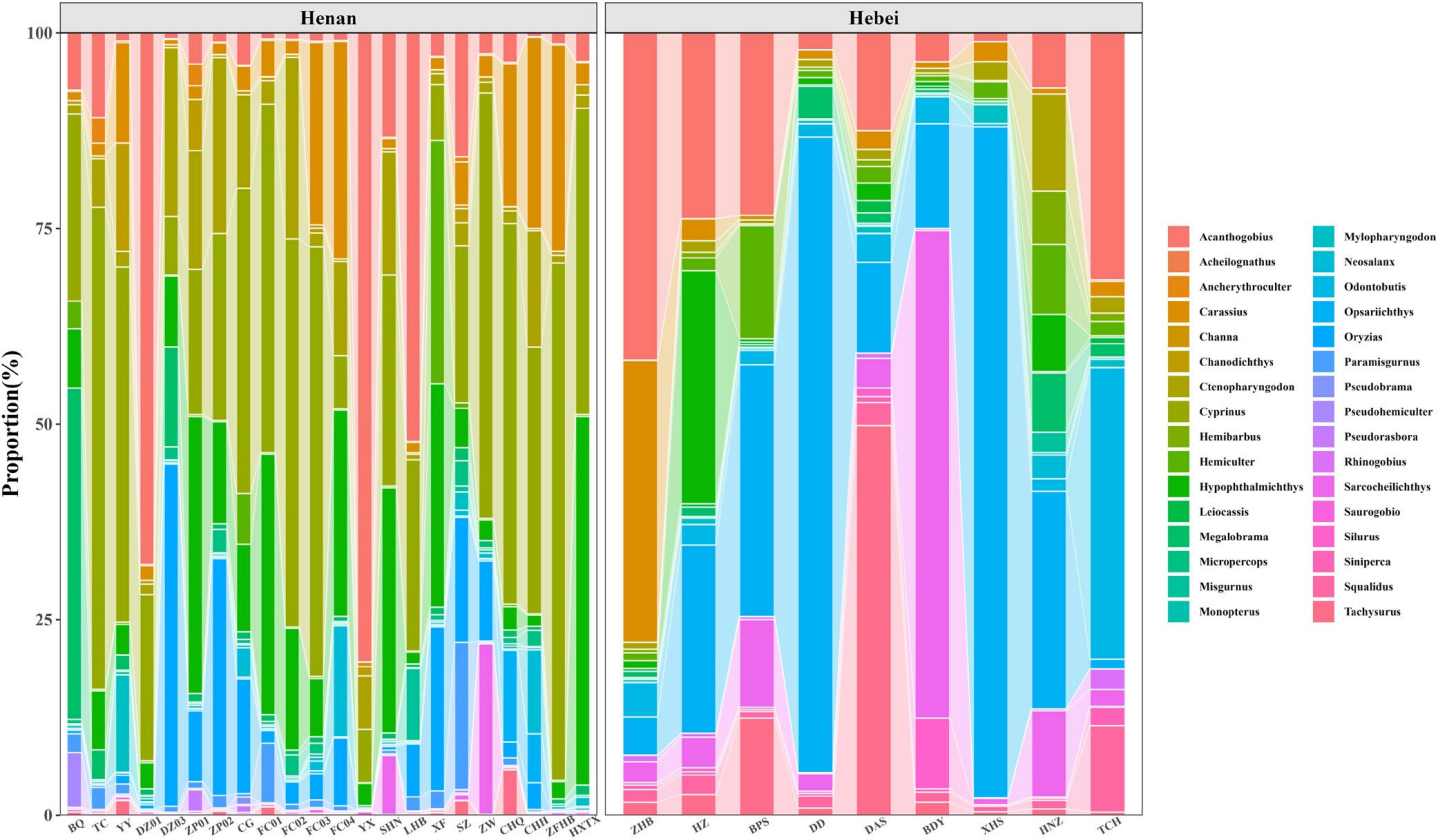
Taxonomic composition and relative eDNA read abundance in the Henan and Hebei provinces of the Middle Route of the South‐to‐North Water Diversion Project.

Through eDNA monitoring, a total of 39 fish taxa belonging to 6 orders, 11 families, and 32 genera were identified in the Middle Route of the South‐to‐North Water Diversion Project. Among these, Cyprinidae dominated with the highest species count (22 taxa, accounting for 56.0%), followed by Bagridae (4 taxa, Table S1). Regarding species richness, 22 species were identified in the Henan province and 23 in the Hebei province. While both regions exhibited comparable levels of taxonomic diversity, their taxonomic compositions differed significantly. Based on relative eDNA read abundance, the fish community in Henan province was characterized by higher proportions of *Cyprinus*, whereas *Opsariichthys* and *Rhinogobius* were more prevalent in Hebei province (Figure 2).

**FIGURE 3:**
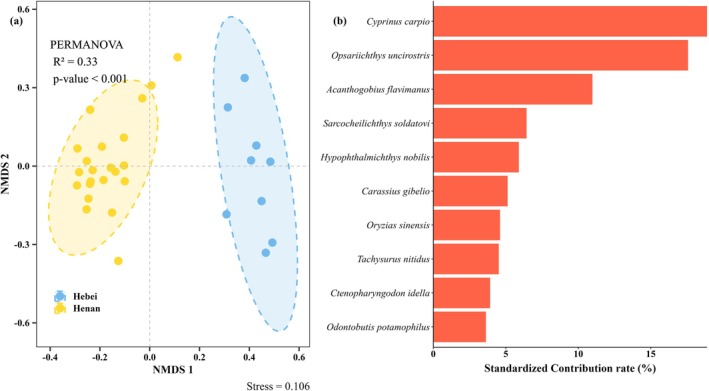
Regional differences in fish community structure along Middle Route of the South‐to‐North Water Diversion Project (a) and the key species contributing to these dissimilarities (b).

NMDS analysis revealed significant spatial heterogeneity in the fish community structure along the main canal of the Middle Route (Stress = 0.14). PERMANOVA confirmed highly significant differences in taxonomic composition between the Henan and Hebei provinces (*p* < 0.001, Figure 3a, Table S3). *Cyprinus carpio* , *Opsariichthys bidens* , and *Rhinogobius flavoventris* were the key species driving community differences between northern and southern sampling sites, together accounting for over 40% of the cumulative contribution to community composition (Figure 3b).

### 3.2 Spatial Trends in Fish Functional Traits

**FIGURE 4:**
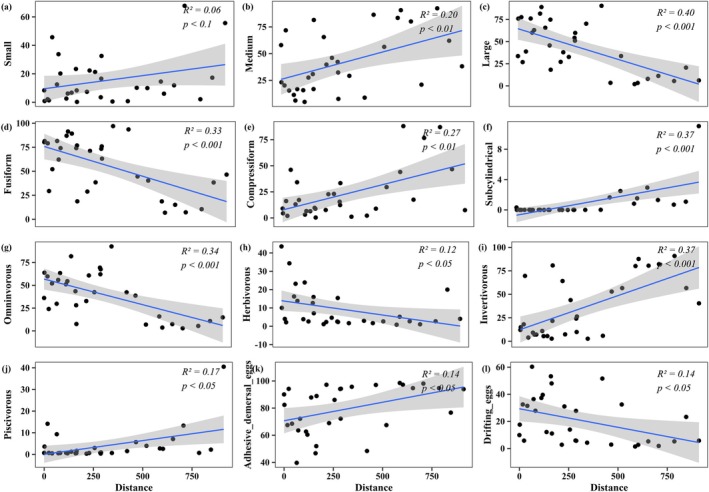
Spatial trends in key functional traits of fish assemblages along the south–north gradient of the main canal. (a–c) Body size (large, medium, and small); (d–f) Body shape (fusiform, compressiform, and subcylindrical); (g–j) Feeding (omnivorous, herbivorous, invertivorous, and piscivorous); (k, l) Spawning type (adhesive demersal eggs and drifting eggs).

Community Weighted Mean (CWM) analysis revealed a distinct north–south succession trend. With increasing distance from the Taocha Headwork, fish body size exhibited a significant reduction. Regarding feeding group composition, the abundance of herbivorous and omnivorous fish decreased markedly from south to north, while invertivorous and piscivorous fish became progressively more prevalent. Additionally, spawning type also diverged between the southern and northern reaches: southern species predominantly employed drifting eggs, while northern species favored adhesive demersal eggs (Figure 4).

**FIGURE 5:**
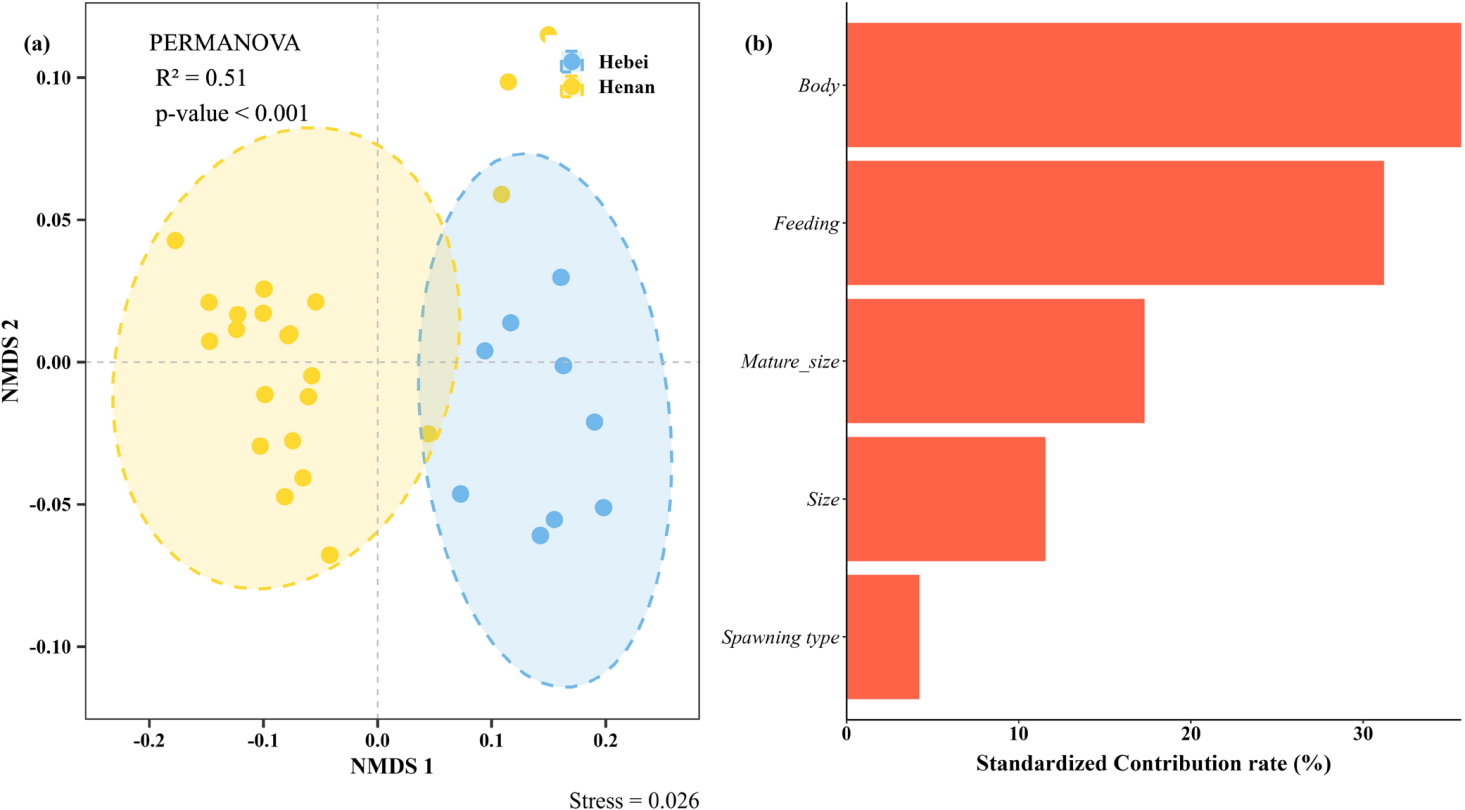
Regional differences in fish functional traits along the Middle Route of the South‐to‐North Water Diversion Project (a) and the key traits driving these dissimilarities (b).

Regional comparisons revealed significant differences between the Henan and Hebei provinces in the NMDS analysis based on functional traits (*p* < 0.001, Figure 5a), with body size and feeding group being the primary drivers of functional dissimilarity (Figure 5b).

### 3.3 Spatial Patterns and Drivers of Fish Beta Diversity

**FIGURE 6:**
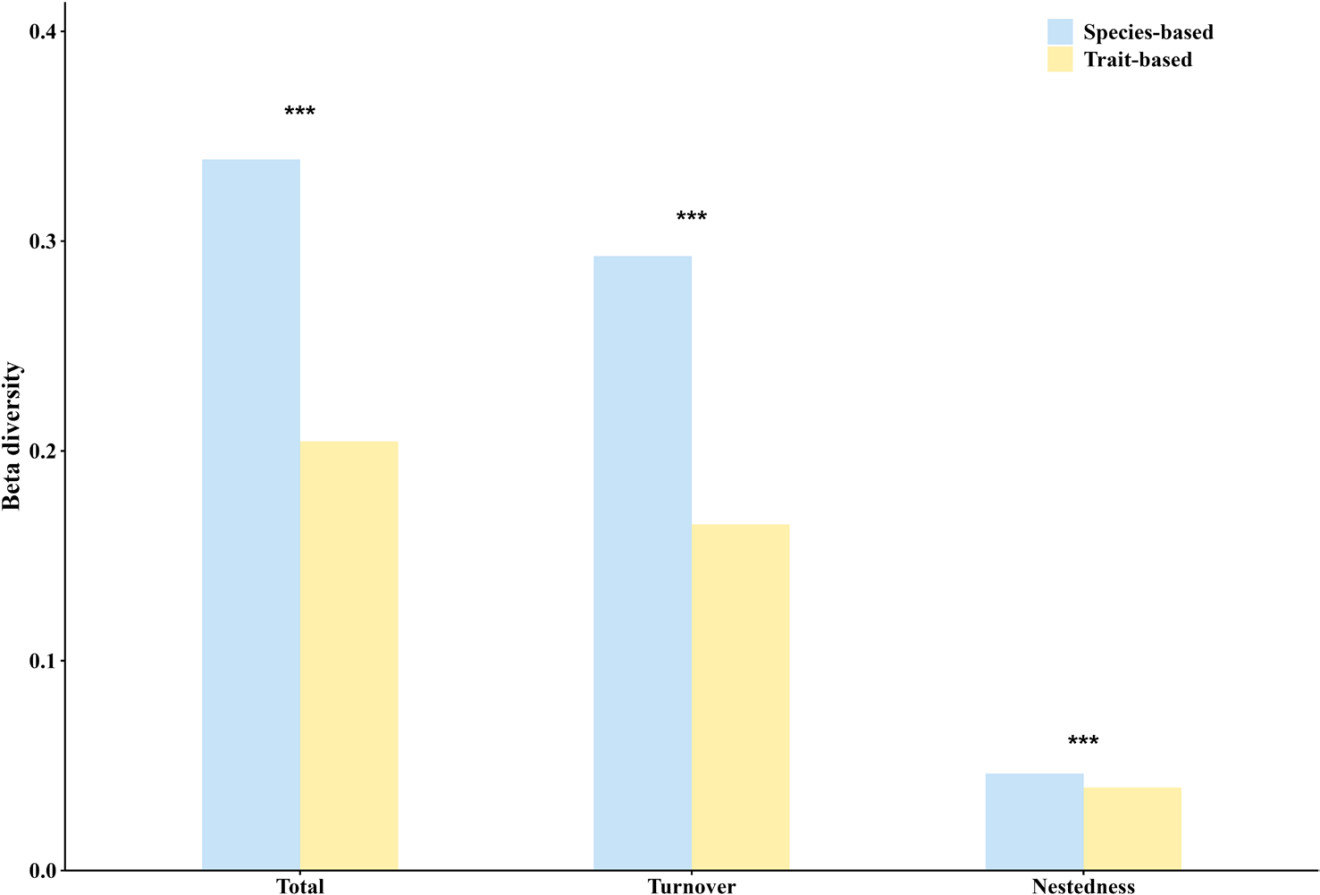
Partitioning of taxonomic and functional beta diversity into turnover and nestedness components for fish assemblages along the Middle Route of the South‐to‐North Water Diversion Project.

**FIGURE 7:**
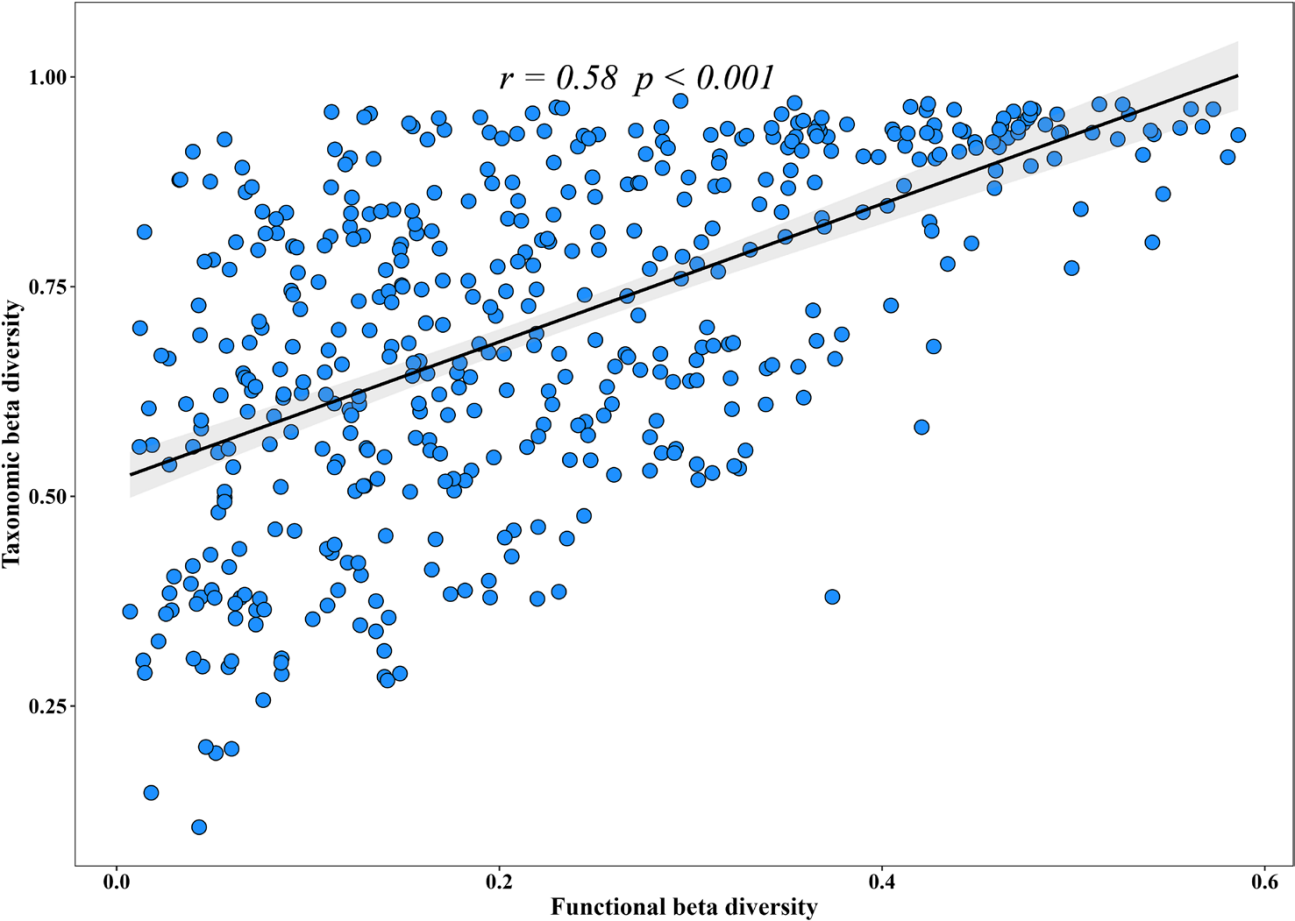
Correlation between taxonomic and functional beta diversity in fish assemblages.

Analysis of fish beta diversity revealed that both taxonomic and functional beta diversity remained at low levels, with values of 0.33 and 0.20, respectively (Figure 6). A significant positive correlation was detected between taxonomic and functional beta diversity (*r* = 0.58, *p* < 0.001, Figure 7); however, functional beta diversity was slightly but significantly lower than its taxonomic counterpart (*p* < 0.001). This numerical lag suggests that spatial turnover in taxonomic composition was not accompanied by an equivalent shift in functional trait composition.

Partitioning of beta diversity revealed that turnover dominated community assembly in both dimensions. Turnover accounted for 86% of taxonomic beta diversity and 80% of functional beta diversity, whereas nestedness contributed only 14% and 20%, respectively (Figure 6). These findings indicate that the compositional differences observed between the southern and northern reaches of the Middle Route arise primarily from the replacement of species and their associated traits, rather than from a simple loss of species richness.

**FIGURE 8:**
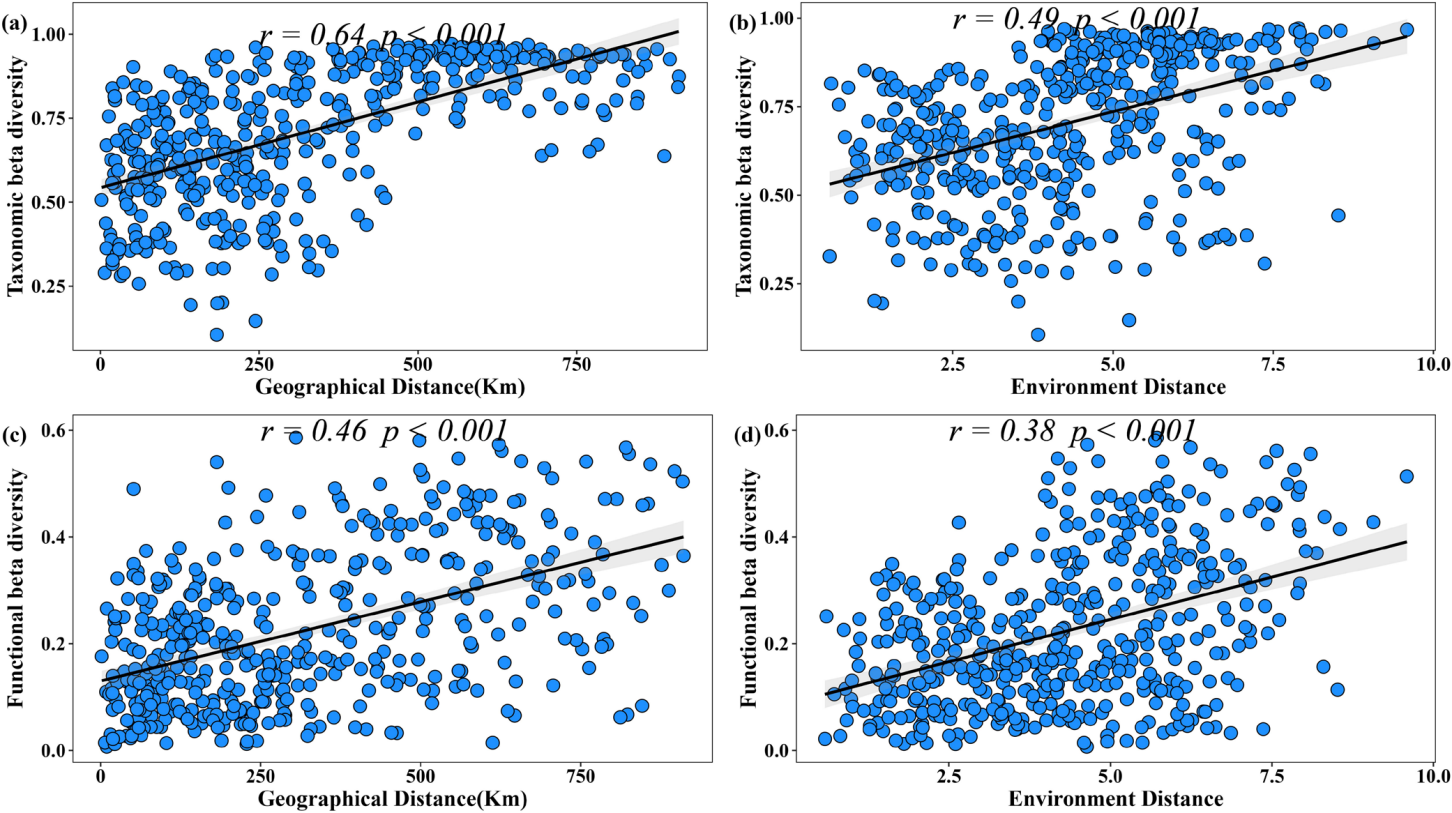
Mantel test revealing the relationships of fish taxonomic and functional beta diversity with geographical and environmental distances: (a) taxonomic beta diversity vs. geographical distance; (b) taxonomic beta diversity vs. environmental distance; (c) functional beta diversity vs. geographical distance; (d) functional beta diversity vs. environmental distance.

The relationship between key factors and fish beta diversity was analyzed using the Mantel test (Table S4). Results indicated that taxonomic beta diversity was significantly and positively correlated with both geographical distance (*r* = 0.64, *p* < 0.001, Figure 8a) and environmental distance (*r* = 0.49, *p* < 0.001, Figure 8b). Functional beta diversity also exhibited significant positive correlations with geographical distance (*r* = 0.46, *p* < 0.001, Figure 8c) and environmental distance (*r* = 0.38, *p* < 0.001, Figure 8d).

**FIGURE 9:**
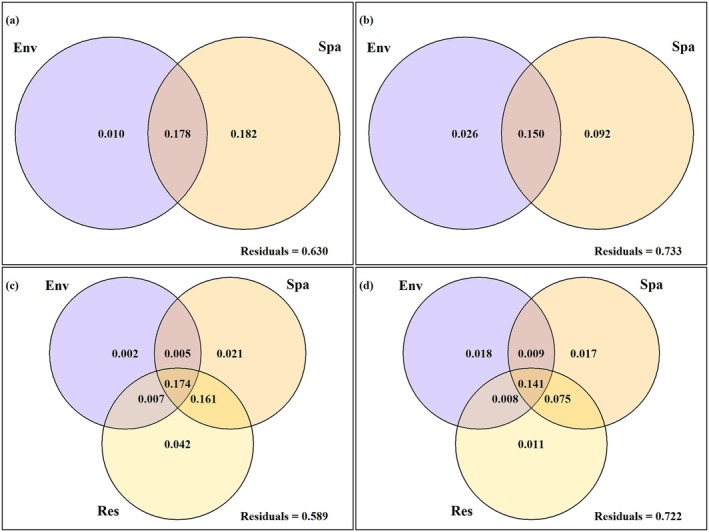
Results of variation partitioning analysis quantifying the contributions of environmental, spatial, and food resource factors to fish beta diversity. (a) Contributions of environmental and spatial factors to taxonomic beta diversity; (b) Contributions of environmental and spatial factors to functional beta diversity; (c) Contributions of environmental, spatial, and food resource factors to taxonomic beta diversity; (d) Contributions of environmental, spatial, and food resource factors to functional beta diversity. Numbers in each Venn diagram indicate the percentage of explained variation (adjusted *R* ^2^) attributable to each factor set and their interactions. Unexplained variation is represented by residuals.

Variation partitioning based on multiple regression on distance matrices (MRM) was used to quantify the relative contributions of spatial factors, environmental variables, and resource availability to fish community assembly. For taxonomic beta diversity, spatial factors exhibited a substantial independent effect (18%), far exceeding that of environmental variables (1%), indicating that environmental effects, partly co‐varying with space, may still contribute to community structure (Table S5). However, the shared fraction between spatial and environmental factors also accounted for a considerable proportion of the variation (18%, Figure 9a). When food resource availability was incorporated into the model, the total explained variation increased to 41%, with resource factors contributing a significant independent effect (4%, Figure 9b, Table S6).

For functional beta diversity, spatial factors again emerged as the primary driver, although the independent contribution of food resources was somewhat diminished compared with that observed for taxonomic beta diversity. Notably, a strong shared effect was detected between spatial factors and resource factors, pointing to the synergistic interplay between dispersal limitation and resource‐associated effects in shaping fish diversity patterns within this artificial canal ecosystem (Figure 9c,d).

## 4 Discussion

### 4.1 Spatial Divergence and Functional Succession of Fish Community Structure

NMDS analysis revealed significant differences in fish community structure between the Henan and Hebei provinces, confirming the reshaping effect of inter‐basin water diversion projects as artificial corridors on fish distribution patterns (Liang et al. 2025; Yan et al. 2025; Zhang, Bi, et al. 2023). As a typical artificially regulated ecosystem, the Middle Route main canal exhibits highly homogenized habitat characteristics: “the entire channel is fully lined with concrete, with a uniformly controlled longitudinal slope, and relatively constant water depth and flow velocity” (Nong et al. 2020); “the main canal is constructed entirely of reinforced concrete and is not connected to external water systems, forming an independent artificial ecosystem” (Ma et al. 2026). This homogenized habitat limits spatial variation in species distribution, thereby maintaining an overall low beta diversity level. Differences in the relative abundances of *Cyprinus carpio* , *Opsariichthys bidens* , and *Rhinogobius flavoventris* between the southern and northern reaches emerged as key drivers of the observed regional differentiation. From a functional trait perspective, the fish assemblages exhibited a clear successional gradient from south to north: with increasing latitude, mean body size tended to decrease, and the trophic structure shifted from omnivores towards piscivores and invertivores. This spatial gradient in functional traits reflects the adaptive responses of fish communities to habitat heterogeneity and resource availability along the canal (Messmer et al. 2011; Tootell and Steele 2016).

Further decomposition of beta diversity revealed that both taxonomic and functional components were relatively low in magnitude, with turnover overwhelmingly dominating over nestedness (accounting for 86% and 80%, respectively). This pattern diverges markedly from the “high species turnover, low functional turnover” observed in most natural rivers (Liu and Wang 2018; Villeger et al. 2013). This indicates that community variation among sampling sites along the main canal stems primarily from the replacement of species and their associated functional traits, rather than from gradients in species richness. Notably, functional beta diversity was significantly lower than its taxonomic counterpart. Following the framework proposed by Villeger et al. (2013), this pattern may indicate functional convergence under highly homogenized artificial systems and could have implications for functional redundancy, although functional redundancy was not directly quantified in this study. Moreover, the predominance of turnover in both taxonomic and functional beta diversity along the Middle Route implies that species replacement across regions is accompanied by a concomitant shift in functional trait composition. Such synchrony may reduce functional redundancy, potentially undermining community stability and resilience (de Bello et al. 2009). In the face of stochastic disturbances, the functional stability of this artificial ecosystem may therefore be increasingly vulnerable.

### 4.2 Drivers of Dispersal Limitation in Homogenized Habitats

Regarding the driving mechanisms of community assembly, this study found that spatial factors (geographic distance) explained a significantly larger proportion of variation than the measured environmental factors. This conclusion challenges the traditional paradigm of “environmental filtering dominance” in natural river research (Heino et al. 2017; Lopez‐Delgado et al. 2020). This divergence likely stems primarily from the highly artificial habitat attributes of the Middle Route: extensive concrete lining and stringent water quality regulation limit the development of environmental gradients, leading to highly homogenized habitats that weaken the intensity of environmental filtering.

Under such circumstances, dispersal limitation with geographical distance as its core proxy emerges as the primary deterministic process shaping fish diversity patterns. Body size, a well‐established surrogate for dispersal capacity (Costello et al. 2015), exhibited a clear latitudinal gradient: larger‐bodied species predominated in the Henan province, whereas smaller‐bodied species characterized the Hebei province. This spatial trend further corroborates the role of dispersal‐based processes in community assembly. Although the independent contribution of environmental factors was modest, spatial factors alone explained more variation in both taxonomic and functional beta diversity than did environmental variables alone, with substantial shared effects between the two. This pattern contrasts with the environmentally driven assembly commonly reported in most natural river studies (Heino et al. 2017; Lopez‐Delgado et al. 2020; Messmer et al. 2011). The extensive spatial extent of our study area (> 1000 km) may have further amplified the relative contribution of spatial factors. Nevertheless, the significant interaction between spatial and environmental factors (Space × Environment) indicates that community assembly in the main canal is not governed by a single process. Overall, spatial structure had a stronger independent contribution than measured environmental variables, but environmental gradients and their spatial covariation may still influence fish beta diversity patterns. These findings underscore the need, when assessing the ecological impacts of large‐scale water infrastructure projects, to pay close attention to how artificial corridors reshape dispersal pathways and modulate the role of stochastic processes in community assembly.

### 4.3 Resource‐Associated Effects on Community Assembly

After incorporating food resources (macroinvertebrates, plankton, and benthic algae) into the analysis, the explanatory power for taxonomic beta diversity increased, with the independent contribution of food resources surpassing that of conventional physicochemical water variables. This result is consistent with bottom‐up resource mediation, whereby the availability of basal resources may play an important role in shaping the spatial distribution of higher trophic‐level consumers such as fish (Macreadie et al. 2010; Tootell and Steele 2016).

Consistent with this view, the elevated abundance of invertivorous fishes observed in the Hebei province was associated with the greater availability of benthic resources in that habitat. In contrast, the explanatory power of food resources for functional beta diversity was comparatively limited, likely reflecting the evolutionary conservatism of traits such as reproductive strategy and body size, which may not be tightly coupled to short‐term trophic interactions. The strong interactive effect detected between spatial factors and food resources further suggests a potential synergistic mechanism: geographical isolation not only directly limits fish spatial dispersal but also indirectly reshapes the trophic structure of assemblages by modulating the spatial heterogeneity of prey organisms.

It is important to acknowledge that the Mantel test, multiple regression on distance matrices (MRM), and variation partitioning employed in this study are exploratory tools that quantify statistical associations but do not establish causal mechanisms. Specifically, these distance‐based approaches cannot fully disentangle the effects of pure spatial processes (e.g., dispersal limitation) from those of spatially structured environmental variation (e.g., water temperature or nutrient gradients that co‐vary with geography). Consequently, our inference that dispersal limitation is a dominant deterministic process should be considered as the best interpretation of the observed patterns, rather than a definitive causal conclusion. Future studies integrating more explicit spatial methods (e.g., distance‐based redundancy analysis, db‐RDA; Moran's eigenvector maps, dbMEM) along with direct measurements of dispersal capacity and hydraulic/habitat modeling are needed to further resolve the relative roles of deterministic vs. stochastic processes in shaping fish assemblages along the Middle Route of the South‐to‐North Water Diversion Project.

## 5 Conclusion

This study indicates that fish community assembly in the artificial ecosystem of the Middle Route of the South‐to‐North Water Diversion Project is associated more strongly with dispersal limitation than environmental filtering. This finding deviates from the deterministic patterns commonly observed in natural river ecosystems. Multidimensional analysis shows that functional beta diversity significantly lags behind taxonomic beta diversity in spatial turnover, exhibiting high turnover rates but suggesting reduced functional redundancy. This suggests that the extensive habitat homogenization inherent in large‐scale inter‐basin water transfers promotes functional convergence among species, potentially undermining the ecological resilience of these artificial corridors. Furthermore, by incorporating food resource availability (particularly macroinvertebrates and plankton) as key mediating factors, our findings suggest that resource‐mediated assembly processes may play an important role in simplified ecosystems. These insights imply that biodiversity management in artificial water transfer systems should shift from conventional water quality monitoring towards an integrated framework encompassing targeted biosecurity surveillance and functional habitat compensation at critical nodes. Conservation strategies should prioritize maintaining the stability of food web structures and implementing habitat enhancement measures at key locations to support the persistence of diverse functional groups, thereby safeguarding the long‐term ecological integrity and water supply security of this major water diversion project.

## Author Contributions

**Zhibin Song:** conceptualization (equal), data curation (equal), formal analysis (lead), writing – original draft (lead). **Jia Li:** writing – review and editing (equal). **Min Zhang:** conceptualization (equal), writing – review and editing (lead). **Zhihai Li:** writing – review and editing (equal). **Zihua Qu:** writing – review and editing (equal). **Haohan Yuan:** writing – review and editing (equal). **Xiaodong Qu:** writing – review and editing (equal). **Baohang Zhang:** data curation (equal), investigation (equal). **Chengwei Chen:** investigation (equal). **Xiaobo Liu:** writing – review and editing (lead).

## Funding

This work was supported by National Key Research and Development Program of China, 2024YFC3213800, 2023YFC3207802. State Key Laboratory of Water Cycle and Water Security, SKL2025RCPY07, SKL2025TDGG01.

## Conflicts of Interest

The authors declare no conflicts of interest.

## Supporting information


**Data S1:** eDNA assignment.

**Data S2:** Environmental variables.

**Data S3:** Fish functional trait matrix.

**Data S4:** Read abundance per site.

**Data S5:** Resource variables_benthic algae.

**Data S6:** Resource variables_macroinvertebrates.

**Data S7:** Resource variables_phytoplankton.

**Data S8:** Resource variables_zooplankton.

**Data S9:** Sequencing‐depth and read‐retention statistics.

**Table S1:** Detected fish taxa list.
**Table S2:** Environmental‐variable ranges.
**Table S3:** The PERMANOVA analysis results.
**Table S4:** Mantel test results.
**Table S5:** Results of variation partitioning between Fish Beta Diversity and Dissimilarity Matrices of Environmental Factors, and Spatial Factors Factors in the Main Channel of the Middle Route of the South‐to‐North Water Diversion Project.
**Table S6:** Results of variation partitioning between Fish Beta Diversity and Dissimilarity Matrices of Environmental Factors, Spatial Factors, and Food Resource Factors in the Main Channel of the Middle Route of the South‐to‐North Water Diversion Project.

## Data Availability

All data supporting this study have been deposited in the Dryad Digital Repository. The data are currently accessible for peer review and will be publicly released upon formal publication of the article. The data are available at: https://doi.org/10.5061/dryad.573n5tbpq.
