# Supplementary material for: Spatial Factors Outweigh Environmental Variables in Explaining Fish Beta Diversity Within A Massive Anthropogenically Homogenized Water‐Transfer Canal
Source: Ecol Evol. 2026 Jul 29;16(8):e74077. doi: 10.1002/ece3.74077 (PMC13416753; doi:10.1002/ece3.74077)
Supplement: Supplementary file 10 — Table S1: Detected fish taxa list. Table S2: Environmental‐variable ranges. Table S3: The PERMANOVA analysis results. Table S4: Mantel test results. Table S5: Results of variation partitioning between Fish Beta Diversity and Dissimilarity Matrices of Environmental Factors, and Spatial Factors Factors in the Main Channel of the Middle Route of the South‐to‐North Water Diversion Project. Table S6: Results of variation partitioning between Fish Beta Diversity and Dissimilarity Matrices of Environmental Factors, Spatial Factors, and Food Resource Factors in the Main Channel of the Middle Route of the South‐to‐North Water Diversion Project. [file ECE3-16-e74077-s009.docx]

**Supplementary Material**

Table S1 detected fish taxa list

| **Number** | **Order** | **Family** | **Genus** | **Species** |
| --- | --- | --- | --- | --- |
| 1 | Osmeriformes | Salangidae | Neosalanx | *Neosalanx taihuensis* |
| 2 | Cyprinodontiformes | Adrianichthyidae | Oryzias | *Oryzias sinensis* |
| 3 | Anabantiformes | Channidae | Channa | *Channa argus* |
| 4 | Synbranchiformes | Synbranchidae | Monopterus | *Monopterus albus* |
| 5 | Siluriformes | Bagridae | Leiocassis | *Leiocassis longirostris* |
| 6 | Siluriformes | Bagridae | Tachysurus | *Tachysurus eupogon* |
| 7 | Siluriformes | Bagridae | Tachysurus | *Tachysurus nitidus* |
| 8 | Siluriformes | Bagridae | Tachysurus | *Tachysurus fulvidraco* |
| 9 | Siluriformes | Siluridae | Silurus | *Silurus asotus* |
| 10 | Anabantiformes | Odontobutidae | Micropercops | *Micropercops swinhonis* |
| 11 | Anabantiformes | Odontobutidae | Odontobutis | *Odontobutis obscura* |
| 12 | Anabantiformes | Gobiidae | Acanthogobius | *Acanthogobius flavimanus* |
| 13 | Anabantiformes | Gobiidae | Rhinogobius | *Rhinogobius cliffordpopei* |
| 14 | Anabantiformes | Sinipercidae | Siniperca | *Siniperca obscura* |
| 15 | Cypriniformes | Cyprinidae | Carassius | *Carassius gibelio* |
| 16 | Cypriniformes | Cyprinidae | Cyprinus | *Cyprinus carpio* |
| 17 | Cypriniformes | Cyprinidae | Chanodichthys | *Chanodichthys erythropterus* |
| 18 | Cypriniformes | Cyprinidae | Ancherythroculter | *Ancherythroculter nigrocauda* |
| 19 | Cypriniformes | Cyprinidae | Mylopharyngodon | *Mylopharyngodon piceus* |
| 20 | Cypriniformes | Cyprinidae | Hemiculter | *Hemiculter leucisculus* |
| 21 | Cypriniformes | Cyprinidae | Pseudobrama | *Pseudobrama simoni* |
| 22 | Cypriniformes | Cyprinidae | Megalobrama | *Megalobrama amblycephala* |
| 23 | Cypriniformes | Cyprinidae | Megalobrama | *Megalobrama terminalis* |
| 24 | Cypriniformes | Cyprinidae | Megalobrama | *Megalobrama pellegrini* |
| 25 | Cypriniformes | Cyprinidae | Hypophthalmichthys | *Hypophthalmichthys nobilis* |
| 26 | Cypriniformes | Cyprinidae | Hypophthalmichthys | *Hypophthalmichthys molitrix* |
| 27 | Cypriniformes | Cyprinidae | Ctenopharyngodon | *Ctenopharyngodon idella* |
| 28 | Cypriniformes | Cyprinidae | Opsariichthys | *Opsariichthys bidens* |
| 29 | Cypriniformes | Cyprinidae | Pseudohemiculter | *Pseudohemiculter* sp. |
| 30 | Cypriniformes | Cyprinidae | Chanodichthys | *Chanodichthys mongolicus* |
| 31 | Cypriniformes | Cyprinidae | Hemibarbus | *Hemibarbus maculatus* |
| 32 | Cypriniformes | Cyprinidae | Pseudorasbora | *Pseudorasbora parva* |
| 33 | Cypriniformes | Cyprinidae | Saurogobio | *Saurogobio dabryi* |
| 34 | Cypriniformes | Cyprinidae | Squalidus | *Squalidus gracilis* |
| 35 | Cypriniformes | Cyprinidae | Sarcocheilichthys | *Sarcocheilichthys wolterstorffi* |
| 36 | Cypriniformes | Cyprinidae | Acheilognathus | *Acheilognathus chankaensis* |
| 37 | Cypriniformes | Cobitidae | Misgurnus | *Misgurnus anguillicaudatus* |
| 38 | Cypriniformes | Cobitidae | Misgurnus | *Misgurnus mizolepis* |
| 39 | Cypriniformes | Cobitidae | Paramisgurnus | *Paramisgurnus dabryanus* |

Table S2 Environmental-variable ranges

| Environmental Factors | Range | Mean | SD |
| --- | --- | --- | --- |
| WT | 14.278 – 28.400 | 20.61 | 5.09 |
| DO | 5.280 – 10.580 | 9.03 | 1.65 |
| Sal | 0.100 – 0.150 | 0.14 | 0.01 |
| pH | 8.000 – 8.810 | 8.36 | 0.21 |
| V | 0.024 – 0.413 | 0.17 | 0.09 |
| CODMn | 1.700 – 2.300 | 1.94 | 0.13 |
| TN | 0.956 – 1.780 | 1.33 | 0.2 |
| TP | 0.021 – 0.180 | 0.07 | 0.05 |
| NH₃-N | 0.025 – 0.230 | 0.04 | 0.04 |
| NO₃⁻-N | 0.750 – 0.950 | 0.88 | 0.04 |
| SiO₃²⁻-Si | 2.000 – 5.800 | 4.9 | 1.09 |
| TOC | 4.000 – 7.200 | 5.21 | 0.71 |

Table S3 The PERMANOVA analysis results

| **Community type** | **Distance matrix** | **R²** | **p-value** |
| --- | --- | --- | --- |
| Taxonomic community | Bray–Curtis | 0.33 | < 0.001 |
| Functional community | Bray–Curtis | 0.51 | < 0.001 |

Table S4 Mantel test results

| **Comparison** | **Mantel r** | **p-value** |
| --- | --- | --- |
| Taxonomic β‑diversity vs. geographic distance | 0.64 | < 0.001 |
| Taxonomic β‑diversity vs. environmental dissimilarity | 0.49 | < 0.001 |
| Functional β‑diversity vs. geographic distance | 0.46 | < 0.001 |
| Functional β‑diversity vs. environmental dissimilarity | 0.38 | < 0.001 |

Table S5 Results of variation partitioning between Fish Beta Diversity and Dissimilarity Matrices of Environmental Factors, and Spatial Factors Factors in the Main Channel of the Middle Route of the South-to-North Water Diversion Project.

| **Explanatory variables / Response variables** | | **Taxonomic β‑diversity** | **Functional β‑diversity** |
| --- | --- | --- | --- |
| Unique | Environmental factors | 0.010 | 0.026 |
|  | Spatial factors | 0.182 | 0.092 |
| Shared | | 0.178 | 0.150 |
| Residual | | 0.630 | 0.733 |

Table S6 Results of variation partitioning between Fish Beta Diversity and Dissimilarity Matrices of Environmental Factors, Spatial Factors, and Food Resource Factors in the Main Channel of the Middle Route of the South-to-North Water Diversion Project.

| **Explanatory variables / Response variables** | | **Taxonomic β‑diversity** | **Functional β‑diversity** |
| --- | --- | --- | --- |
| Unique | Environmental factors | 0.002 | 0.018 |
|  | Spatial factors | 0.021 | 0.017 |
|  | Resource factors | 0.042 | 0.011 |
| Shared | Env∩Spa | 0.005 | 0.009 |
|  | Env∩Res | 0.007 | 0.008 |
|  | Spa∩Res | 0.161 | 0.075 |
|  | Env∩Spa∩Res | 0.174 | 0.141 |
| Residual | | 0.589 | 0.722 |
